# Effects of multiple vein microjoints on the mechanical behaviour of dragonfly wings: numerical modelling

**DOI:** 10.1098/rsos.150610

**Published:** 2016-03-23

**Authors:** H. Rajabi, N. Ghoroubi, A. Darvizeh, E. Appel, S. N. Gorb

**Affiliations:** 1 Zoological Institute, Functional Morphology and Biomechanics, Kiel University, Kiel, Germany; 2 Department of Mechanical Engineering, The University of Guilan, Rasht, Iran; 3 Young Researchers Club, Rasht Branch, Islamic Azad University, Rasht, Iran

**Keywords:** insect flight, dragonfly wing, vein joint, finite-element modelling, resilin

## Abstract

Dragonfly wings are known as biological composites with high morphological complexity. They mainly consist of a network of rigid veins and flexible membranes, and enable insects to perform various flight manoeuvres. Although several studies have been done on the aerodynamic performance of Odonata wings and the mechanisms involved in their deformations, little is known about the influence of vein joints on the passive deformability of the wings in flight. In this article, we present the first three-dimensional finite-element models of five different vein joint combinations observed in Odonata wings. The results from the analysis of the models subjected to uniform pressures on their dorsal and ventral surfaces indicate the influence of spike-associated vein joints on the dorsoventral asymmetry of wing deformation. Our study also supports the idea that a single vein joint may result in different angular deformations when it is surrounded by different joint types. The developed numerical models also enabled us to simulate the camber formation and stress distribution in the models. The computational data further provide deeper insights into the functional role of resilin patches and spikes in vein joint structures. This study might help to more realistically model the complex structure of insect wings in order to design more efficient bioinspired micro-air vehicles in future.

## 1. Introduction

Insect wings are subjected to a combination of bending and torsional deformations during flight. However, the quality of the deformations varies widely among the species in a specific order or even between those in one suborder [1–3]. Taking into account that wing deformations may significantly influence the flight performance, various flight capabilities are expected from different insect species depending on the wing deformability.

Both dragonflies and damselflies belong to the order Odonata. However, they exhibit very different flight behaviours. Most of the dragonflies are fast fliers with broad-based wings and posterior-distally curved longitudinal veins [4]. They usually perform flapping and sometimes gliding and soaring flight [5,6]. In comparison with damselflies, they have a higher wing beat frequency, and therefore a higher rate of energy consumption in flight [7]. The wings of dragonflies, with limited twisting, are able to produce lift forces only in downstroke [8]. By contrast, damselflies, with their narrow-based and nearly identical fore- and hindwings, are known by their slow flight, low flapping frequency and large wing twisting [9,10]. Damselflies, which are classified as perchers, use both up- and downstrokes for aerodynamic force generation [10].

Many years have passed since the first studies describing the influence of insect wing design on its aerodynamic performance were published [11,12]. The authors revealed that Odonata wings are complex structures which use both active and passive mechanisms to control their deformations in flight. Since Odonata, similar to other insects, have wings supplied with flight muscles only at the base, but not in the central and distal parts, passive deformations due to the wing architecture and its material properties play a much more important role in their flight capabilities. Our current understanding is that morphological adaptations allowing passive control of wing deformations are as follows: (i) venation pattern [13], (ii) venational fractures [12], (iii) vein joints [11], (iv) thickened areas [12], (v) fold and flexion lines [14–16], (vi) material gradients [17–19], and (vii) spikes located in the vicinity of joints [11,17].

So far, many of the mentioned mechanisms producing the passive control of wing deformations have not been incorporated into the engineering design of artificial wings of micro-air vehicles. Considering the existing technological gap between insect wings and artificial wings of our modern flapping robots [20], using mechanisms inspired by dragonflies might lead to the development of novel flapping technical wings with more durability and aerodynamic efficiency.

In Odonata, vein joints, which are distributed through the wings and can be found in the junction of the vast majority of veins, seem to provide (together with the spikes) a major mechanism controlling wing deformations in a more extended area. Vein joints in Odonata are presumably adapted to provide different structural flexibilities in different wing regions. It has been suggested that the deformability of the wing at vein joints is mainly affected by their geometric features [8,11] as well as their material composition [17,19,21,22].

In 2011, Donoughe and co-workers, using a series of experimental tests, showed that some of the vein joints can give more flexibility to the wing structure in specific regions [18]. Their experiments supported the hypothesis to classify the vein joints into two main types of flexible and fused [11] or mobile and immobile [19]. The detailed morphological study of Appel & Gorb [17] on the wings of 22 species of 20 different families of Odonata showed that vein joints are much more complex than was thought before. Using a combination of scanning electron microscopy (SEM), fluorescent light microscopy and confocal laser scanning microscopy techniques, they suggested a new classification for vein joints and their combinations in Odonata and speculated on their possible functional significances.

### 1.1. Combination of the vein joints in Odonata

The previous results obtained from microscopic examinations indicated that according to the geometric characteristics and material compositions, vein joints can be divided into seven main types [17]. (i) Double rigidly fused joints (DRFJ) consist of a cross-vein that is rigidly fused to a longitudinal vein (see the electronic supplementary material, figure S1*a*). Cross-vein in this joint type is located at the same level as the adjacent longitudinal vein and seems to be noticeably elevated from the wing membrane. (ii) Double-fused joints (DFJ) are composed of a cross-vein that is fused to a longitudinal vein at a considerably lower level (see the electronic supplementary material, figure S1*b*). The contact area between longitudinal and cross-veins in the DRFJ is much larger than that of the DFJ of a comparable size. (iii) Flexible-fused joints (FFJR1) are formed by a cross-vein directly fused to an adjacent longitudinal vein at the hill side and indirectly connected to that by means of a resilin patch at the valley side (see the electronic supplementary material, figure S1*d*,*f*). (iv) Double flexible joints (DFJR2) are indirectly connected to a longitudinal vein by means of two resilin patches at both hill and valley sides (see the electronic supplementary material, figure S1*h*). (v) Double flexible joints with resilin patches at both sides and a spike located on the hill side (DFJS1) (see the electronic supplementary material, figure S1*i*,*j*) and (vi) double flexible joints with resilin patches and spikes on both sides (DFJS2) (see the electronic supplementary material, figure S1*k*,*l*) are different from the DFJR2 in the presence of spikes. (vii) Bridge joints (BJ) are made up of two fused cross-veins which lie on an adjacent longitudinal vein (see the electronic supplementary material, figure S1*c*). This type of vein joint is usually rare in the wings of Odonata. Electronic supplementary material, figure S1 presents selected micrographs of the joint types described here.

Wings of Odonata are dominated by combinations of one or two specific vein joint types. Considering the distribution of the vein joints within the wings of 22 species of Odonata examined by Appel & Gorb [17], five groups of wings with different vein joint patterns can be distinguished. (i) Group 1 is characterized by the combination of flexible-fused joints (FFJR1) (table 1). The wings of the dragonfly *Epiophlebia superstes* can be included in this group [17,22]. (ii) Group 2 shows a combination of flexible-fused joints (FFJR1) with double flexible joints (DFJR2) that are usually located at the convex and concave longitudinal veins, respectively (table 1). Such a combination was found in the wings of different species, such as *Aeshna cyanea*, *Ae. constricta*, *Ae. verticalis*, *Phyllopetalia apicalis*, *Stylogomphus suzukii* and *Tanypteryx pryeri* [17,18]. (iii) Group 3 is characterized by both flexible-fused joints (FFJR1) and double flexible joints with spikes located on the hill side (DFJS1). In this group, FFJR1 and DFJS1 are typically found at the convex and concave longitudinal veins, respectively (table 1). The dragonflies *Sympetrum vicinum* and *S. vulgatum* and the damselflies *Copera annulata*, *Psaironeura remissa*, *Microstigma rotundatum* and *Enallagma divagans* belong to Group 3 [17,18]. (iv) Representatives of Group 4 can be distinguished by the occurrence of DFJ at the convex longitudinal veins together with double flexible joints containing spikes on both hill and valley sides (DFJS2) located at the concave longitudinal veins (table 1). *Lestes rectangularis*, *Mortonagrion hirosei*, *Philogenia cassandra*, *Cora cyane*, *Palaemnema clementia*, *Perissolestes romulus* and *Rimanella arcana* are classified in this group [17,18]. (v) The wings in Group 5 are dominated by the presence of double flexible joints with spikes on the hill and valley sides (DFJS2) (table 1). Representatives of this group are *Calopteryx splendens*, *C. angustipennis, Euphaea yayeyamana*, *Mnais pruinosa pruinosa* and *Matrona basilaris basilaris* [17,18]. The red rectangles in the electronic supplementary material, figure S2 correspond to the approximate regions of wings, where the vein joint combinations may occur.

**Table 1.** Characteristics of FE models of vein joint combinations. Symbols ✓ and × indicate the presence and absence of the mentioned feature. Vein joints are numbered from left to right. DFJ, double fused joint; FFJR1, flexible-fused joints; DFJR2, double flexible joints; DFJS1, double flexible joints with a spike located on the hill side; DFJS2, double flexible joints with spikes located on both sides.

two-dimensional view of models							
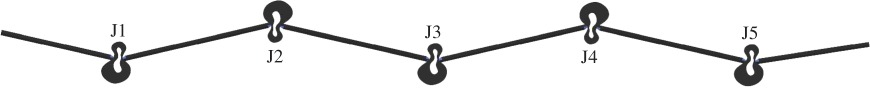							
vein joint group	vein joint no.	vein joint type	hill-sided resilin	valley-sided resilin	hill-sided spike	valley-sided spike	two-dimensional view of the joints
1	J1, J3, J5 (concave joints)	FFJR1	×	✓	×	×	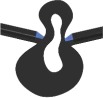
	J2, J4 (convex joints)	FFJR1	×	✓	×	×	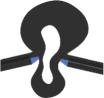
2	J1, J3, J5 (concave joints)	DFJR2	✓	✓	×	×	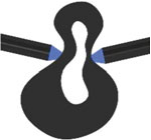
	J2, J4 (convex joints)	FFJR1	×	✓	×	×	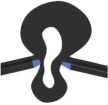
3	J1, J3, J5 (concave joints)	DFJS1	✓	✓	✓	×	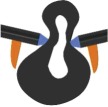
	J2, J4 (convex joints)	FFJR1	×	✓	×	×	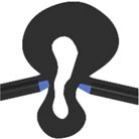
4	J1, J3, J5 (concave joints)	DFJS2	✓	✓	✓	✓	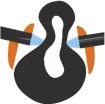
	J2, J4 (convex joints)	DFJ	×	×	×	×	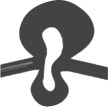
5	J1, J3, J5 (concave joints)	DFJS2	✓	✓	✓	✓	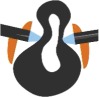
	J2, J4 (convex joints)	DFJS2	✓	✓	✓	✓	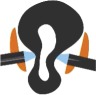

There are several studies available in the literature that employed finite-element (FE) method to numerically model dragonfly wings as a whole. However, given the structural complexities of insect wings, many of the previously developed models contain inevitable huge oversimplifications [23,24]. Therefore, in our opinion, comparative studies on individual wing components may serve as a much more useful approach to understand the role of each single component in the functionality of insect wings.

In our previous work, we performed a comparative numerical study on the deformation experienced by each single vein joint subjected to a bending moment [25]. The obtained data showed how the geometry, the presence of resilin patches and spikes can affect the angular deformation of an isolated joint. However, in a real wing, a single vein joint is surrounded by other joints. How do different combinations of vein joints influence the mechanical behaviour of insect wings including the deformation mechanism, stress distribution and camber formation?

Donoughe and co-workers tried to answer this question by performing mechanical tests on the wings of the damselfly *Enallagma civile* [18]. They measured the angular deformations of the wing around RP2, IR2 and RP3/4 veins subjected to an external rotation. The results showed that the combination of flexible and fused joints in the insect wings affects their degrees of deformation, compared to the situation when they are isolated. Considering the limitations of their experimental tests and the difficulty of performing precise experiments on small wing samples, we decided to undertake the present numerical study, in order to investigate the effect of vein joint combinations on the wing deformation. Based on the previously reported morphological data, we developed FE models of five different vein joint combinations and tested their mechanical behaviour under dorsal and ventral loadings. The aim of this paper is to give a better insight into the function of particular combinations of vein joints in the complex structure of insect wings.

## 2. Material and methods

### 2.1. Finite-element modelling

The commercial FE software ABAQUS/Standard version 6.14 was used to develop three-dimensional models of five main vein joint combinations described in §1.1. In order to investigate the effect of vein joints on the mechanical behaviour of the models, independently from the other parameters, the same geometric characteristics, such as size, corrugation angles, vein radius and membrane thickness were assigned to all models. In such a comparative study, this modelling strategy is very important to eliminate the effects induced by other factors, except the geometry and material composition of the joints.

The FE models of single vein joints have been previously developed based on the microscopic data taken from our previous studies [17,22,25]. The dimensional measurements on the micrographs were performed using GIMP 2.8.10 (GNU Image Manipulation Program, authored by Peter Mattis and Spencer Kimball; http://www.gimp.org/). The results from measurements included detailed information about the geometry of the vein joints, their resilin patches and spikes.

The FE models of vein joint combinations were constructed by embedding the models of the single vein joints in a predefined corrugated pattern of the connecting cross-veins and membranes. These models represent a strip of the wings, mainly from the posterior-distal region, which contain five longitudinal veins (but not the leading and trailing edges; see the electronic supplementary material, figure S2). Each model consists of five vein joints, where two of them are located at convex veins and the others at concave ones (table 1). The developed FE vein joint combination models are as follows. (i) Model 1 is representative of the Group 1 and only made up of flexible-fused joints containing resilin patches at their flexible side (FFJR1). (ii) Model 2, representative of the Group 2, consists of a combination of double flexible (DFJR2) and flexible-fused (FFJR1) joints. (iii) Model 3, representative of the Group 3, is composed of double flexible joints with spikes on the hill side (DFJS1) together with flexible-fused joints with resilin patches at the flexible side (FFJR1). (iv) A combination of double flexible joints with spikes on both hill and valley sides (DFJS2) and DFJ is used to develop Model 4 of the Group 4 of vein joint combinations. (v) Model 5 represents the Group 5 and is dominated by the combination of only double flexible joints with spikes on both sides (DFJS2). The characteristics of the models together with the two-dimensional views of the vein joints located at the convex and concave longitudinal veins are given in table 1. The input files of the FE models, used in this study, are available in the electronic supplementary material (Model S1–S5).

The longitudinal veins in all models have the same dimensions. They were measured from SEM images of the anterior radial vein in the right forewing of *E. superstes*. The maximum and minimum external diameters of the longitudinal veins are 121 µm and 22 µm, respectively. The longitudinal veins were connected to each other by a series of identical cross-veins with a length of 457 µm. The membranes connected to the longitudinal and cross-veins were assumed to have a constant thickness of 2 µm through the models [26–28]. Taking into account that the spikes on the hill side of vein joints are usually larger than those on the valley side [17], they were modelled to have different dimensions (hill-sided spikes are about two times larger than those located on the valley side). The corrugation angle, in all models, was chosen to be about 150° [24].

**Figure 1.**
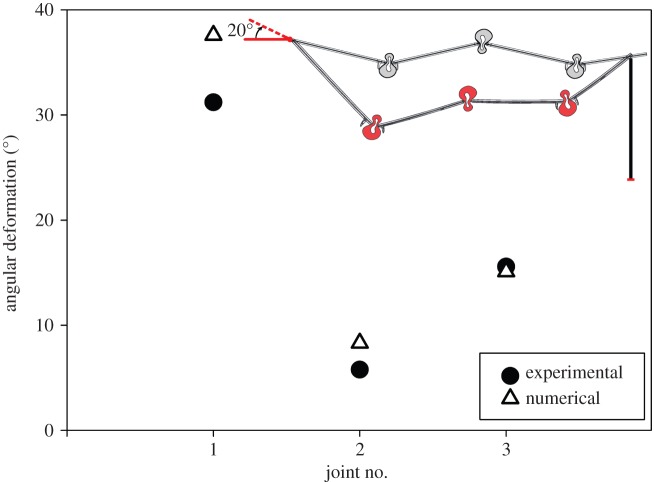
Angular deformation of the vein joints in the model used for the validation analysis. As seen in the top-right sketch, similar to the experiment conducted by Donoughe *et al.* [18], the model is under a 20° angular deformation applied to the free end of the left-sided cross-vein, which is adjacent to the leading edge of the wing. On the right-hand side, the model is fixed with a pin which allows rotation, but prevents the downward displacement. This sketch shows the undeformed and deformed shapes of the model with grey and red colours, respectively. Comparison of the results shows a good agreement between the data obtained from experiments and numerical simulations. Vein joints are numbered from left to right. The length of the linking cross-veins between the vein joints, from left to right, is 487 µm, 457 µm, 457 µm, and 346 µm, respectively.

We developed an additional FE model of the vein joint combinations, to simulate the experimental test performed by Donoughe *et al.* [18]. This model, which is a smaller one from the Group 3 of vein joint combinations, contains a flexible-fused joint (FFJR1) located at the convex longitudinal vein and two double flexible joints with spikes on the hill side (DFJS1) located at the concave longitudinal veins. Similar to the other models, vein joints in this model are connected to each other by linking cross-veins and membranes (see the sketch in the top-right corner of figure 1). The dimensions of the model were selected to be the same as those of the samples that were experimentally examined [18]. The results from the analysis of this model are used to verify the validity of the modelling method and the solving procedure.

### 2.2. Material properties

Our previous numerical simulations indicated that a linear elastic material model can effectively represent the mechanical behaviour of insect wings and their structural elements [25,29–34]. Based on this assumption, the same material properties as those previously used for the simulation of the mechanical behaviour of single vein joints [25] were used in this study.

In a general case, a vein joint combination model is made up of longitudinal veins, cross-veins, adjacent membranes, resilin patches and spikes. Taking into account that there are no experimental data available on the material properties of dragonfly wing veins, a Young's modulus of 3 GPa, which corresponds to the stiffness of the cuticle of the hind tibiae of the locust *Schistocerca gregaria*, was assigned to both longitudinal veins and cross-veins [35]. Based on the experimental data from literature, the membrane of the wing was modelled as an isotropic material with a stiffness of 2.85 GPa [36–38]. The elastic resilin patches were assumed to have an elastic modulus of 1 MPa [39]. Spikes were modelled with the same stiffness as the wing veins. A Poisson's ratio of 0.49 was assigned to all the elements of the models [40].

### 2.3. Loading and boundary conditions

We examined the validity of this modelling method and numerical solution for each single vein joint in our previous work [25]. However, in order to additionally evaluate the computational analysis used in this study, we first simulated the experimental tests performed on the vein joint combinations [18]. For this purpose, the model used in the validation study was located on a fixed insect pin at one side. Then, as shown in the sketch in figure 1, the angular deformation of each vein joint was measured subjected to a rotation of 20° applied to the cross-vein at the free end of the model, which is adjacent to the leading edge of the wing. It is assumed that the insect pin prevents the downward movement of the model at that point.

After the validation study, the mechanical behaviour of the models of five vein joint combination groups was studied under a uniform pressure applied on their dorsal and ventral surfaces. We found that pressures equal to or less than 4.25 Pa and 3.00 Pa on the dorsal and ventral surfaces of the models, respectively, cause no contact between the spikes and their adjacent longitudinal veins. Therefore, these two pressure values were used to analyse the deformation of the models without the direct effect of the spikes. A higher pressure of 60 Pa, which is approximately equal to one half of the pressure exerted by the dragonfly weight on each of its wings (measured for the dragonfly *Sympetrum frequens*) [41], was chosen to achieve a relatively larger deformation and further to investigate the effect of the spikes on the mechanical behaviour of the developed models. This pressure was applied on both dorsal and ventral surfaces of the models.

**Figure 2.**
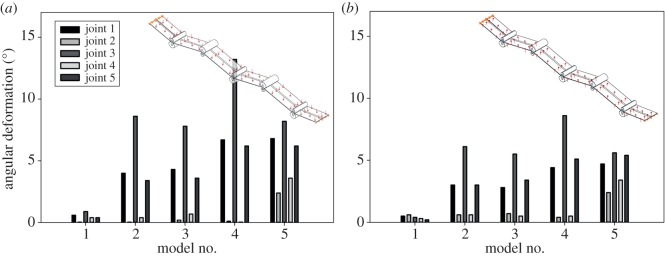
Comparison of the angular deformation of each single vein joint in vein joint combination models before occurrence of physical contacts between spikes and longitudinal veins. Models are under 4.25 Pa pressure (*a*) on the dorsal side and 3 Pa pressure (*b*) on the ventral side. Higher pressures cause a contact between spikes and their adjacent longitudinal veins. Vein joints are numbered from left to right. The movements of the models are fixed in all directions on their left-hand sides and only in the vertical direction on the right-hand side.

In Odonata, ambient veins develop a margin for wings, which is not entirely free to move. This relatively stiff margin, consisting of leading and training edges, serves as a supporting frame for the wing structure. Hence, in our simulations, we fixed the movements of the models in all directions at the side which is adjacent to the leading edge spar of the wing. At the other side, which is close to the trailing edge, the movement of the models is restricted in upward/downward directions. It is highly likely that this boundary condition is a suitable choice to represent the natural rotations of the vein joints as well as the complex mechanical behaviour of the models. The sketches in the top-right corner of figure 2*a*,*b* show the uniform pressures on the dorsal and ventral surfaces of a vein joint combination model, respectively. The associated boundary conditions are shown at both sides of the model.

Taking into account that for large pressures there may be contacts between spikes and their adjacent longitudinal veins, a contact analysis was necessary to define the interaction between the contact surfaces. Considering the estimated accuracy, a surface-to-surface discretization was employed for this purpose [42]. The meeting surfaces of spikes and longitudinal veins were chosen as the master and slave surfaces, respectively. This allows only a small penetration of spikes into adjacent longitudinal veins, but longitudinal veins are constrained not to penetrate into spikes. A linear penalty-based constraint enforcement method was used to approximate the relative motion of the master and slave surfaces. This method, which reduces the computational time, imposes a stiff pressure-overclosure behaviour to the in-contact bodies [42]. According to this approach, the contact force is assumed to be linearly related to the penetration depth.

We conducted a mesh convergence analysis for each model under each loading condition. This analysis was performed by reducing the size of elements and solving the problem again to obtain consistent results which were independent of mesh density. This mesh convergence study was a necessary step to ensure the accuracy of the results.

## 3. Results

The results from the simulation of the experimental torsion test on a section of the wing of the damselfly *E. civile* are illustrated in figure 1. The sketch in the top-right of the figure shows the wing section before and after the applied rotation with the grey and red colours, respectively. The deformation pattern of the model is in very good agreement with that observed experimentally [18]. Based on the simulation results, the first, second and third vein joints of the model undergo a torsional deformation of 37.6°, 8.3° and 15.1°, respectively. The obtained values show a relatively small difference compared to the experimental data.

Figure 2 presents the angular deformation of the vein joints of each model compared to those of the other models. As shown in the sketches in the top-right corner of figure 2*a*,*b*, the models are under 4.25 Pa and 3.00 Pa uniform pressures on the dorsal and ventral surfaces, respectively. Under these loading conditions, no contact was observed between the spikes and longitudinal veins. In both dorsal and ventral loadings, Model 1 which consists of only one-sided resilin joints (FFJR1) demonstrates the smallest overall deformation. Comparison of the angular deformation of the vein joints in this model with their corresponding joints in the other models reveals a considerable difference especially for the joints at the concave longitudinal veins (Joints 1, 3 and 5). The angular deflection of these vein joints in Model 1 is at least 5.6 times and at most 27 times less than those observed in the corresponding vein joints in the other models.

The vein joints located at the concave longitudinal veins in Models 1 and 2 (Joints 1, 3 and 5) are flexible-fused joints with resilin patches at the valley side (FFJR1) and double flexible vein joints containing resilin patches at both sides (DFJR2), respectively. The mentioned vein joints are combined with similar joint types (FFJR1) at the convex longitudinal veins in both models (Joints 2 and 4). However, the comparison of the angular deformation exhibited by these two different vein joint types located at the concave veins in Models 1 and 2 (FFJR1 and DFJR2) shows a considerably greater flexibility provided by double-sided resilin vein joints (DFJR2) in contrast to one-sided vein joints (FFJR1).

Models 2 and 3 are different in the spikes that are located on the concave vein joints in Model 3. Taking into account that no contact occurred between the spikes and longitudinal veins in this loading condition, Models 2 and 3 exhibited almost the same deformation (figure 2). Comparison of the angular deformation of vein joints in these two models shows a noticeably larger deformation in the vein joints located at the concave longitudinal veins than those located at the convex veins (at least 5.1 times for dorsal loading and 4 times for ventral loading).

Considering no spike influence, Models 3 and 4 are different in flexible-fused vein joint (FFJR1) and DFJ at their convex longitudinal veins. Comparison of the angular deformation of these two different joint types in Models 3 and 4 (Joints 2 and 4), in both dorsal and ventral loadings, shows a relatively slight difference. However, interestingly, the angular deformation of double-sided resilin joints at the concave longitudinal veins in Model 4 (Joints 1, 3 and 5) is relatively larger than the deformation experienced by the similar vein joint types in Model 3.

The DFJ located at the convex longitudinal veins (Joints 2 and 4), in Model 4, contain no resilin patches. These rigid joints undergo a relatively small deformation in contrast to the corresponding vein joints in the other models. The difference between the deformations of these joints and the corresponding vein joints at the convex longitudinal veins in Model 5 is clearly visible, although all of them, in both models, are surrounded by the same joint types (DFJS2).

A more uniform deformation pattern was observed in Model 5, compared to the other models, in both dorsal and ventral loadings. As illustrated in figure 2, the double-sided resilin vein joints, located at the convex longitudinal veins in Model 5 (Joints 2 and 4), experience larger angular deformation than the corresponding vein joints in the other models. The angular deformation of these two joints is at least 3.4 times and at most 18.0 times larger than those of the one-sided resilin joints located at the convex sides of Models 1–3. The observed difference is more pronounced between the deformation of the mentioned double-sided resilin joints in Model 5 with those of the rigid vein joints in Model 4 (at least 4.8 times and at most 120.0 times).

**Figure 3.**
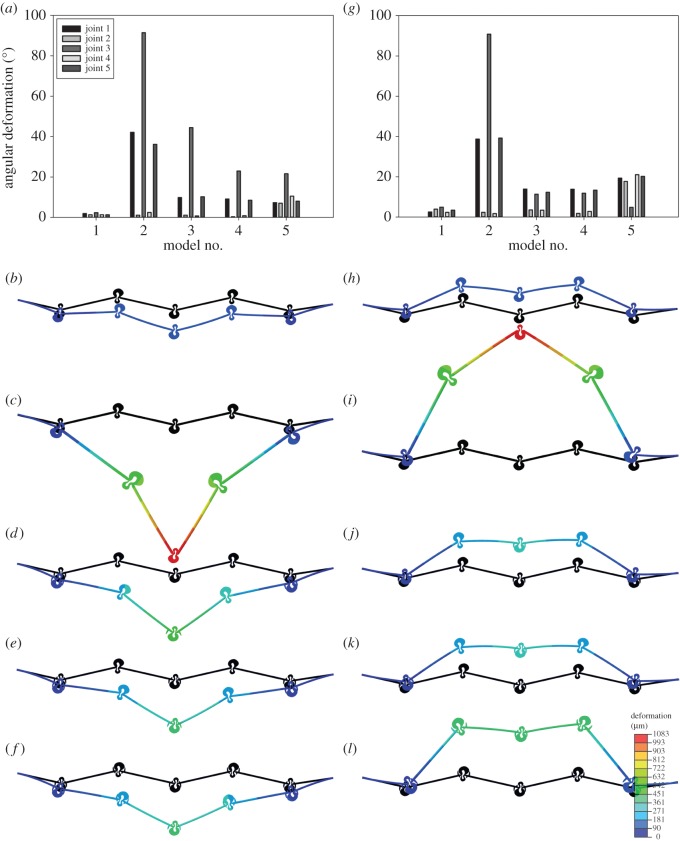
Comparisons of the angular deformation of each single vein joint in vein joint combination models and the overall deformation of each model after occurrence of physical contacts between spikes and longitudinal veins. Models are under a 60 Pa pressure on the (*a*–*f*) dorsal and (*g*–*l*) ventral surfaces. The overall deformation of each model is shown with respect to its original shape and position. Vein joints are numbered from left to right. The movements of the models are fixed in all directions on the left-hand side and only in the vertical direction on the right-hand side.

Figure 3 compares the angular deformations of vein joints in the five developed models subjected to 60 Pa pressure on their dorsal (figure 3*a*–*f*) and ventral (figure 3*g*–*i*) surfaces. The overall deformation of each model with respect to its original position is also presented in this figure. As seen here, even after increasing the pressure applied to the models, which causes physical contact between spikes and longitudinal veins in spike-containing joints (in Models 3–5), Model 1, which consists only of flexible-fused vein joints (FFJR1), exhibits the smallest deformation.

Under the same loading condition, Model 2, which consists of a combination of double flexible (DFJR2) and flexible-fused (FFJR1) vein joints, experiences the largest deformation, compared to all the other models. This large deformation is mainly due to the large angular displacement of double flexible joints (DFJR2), located at concave longitudinal veins (Joints 1, 3 and 5), while the flexible-fused joints (FFJR1) experience only a small deformation.

The physical contacts between the spikes and the concave longitudinal veins, in Model 3, prevented further rotation of cross-veins around the longitudinal veins (see figure 3*d*,*j*). As seen in figure 3*d*,*j*, dorsal loading on Model 3 establishes contacts between the spikes and longitudinal veins in the first and last joints, respectively; however, the contact between the spikes and longitudinal vein in the middle joint occurs only when the model is under ventral loading. The middle vein joint in this model undergoes considerably different deformations in both dorsal and ventral loading conditions.

The smaller spikes, located on the valley sides of double flexible vein joints (DFJS2), together with the rigid DFJ, located at convex longitudinal veins, are two main mechanisms which provide additional rigidity to Model 4 compared to Model 3. As seen in figure 3, the overall deformation of Model 4 is less than that of Model 3, especially in the dorsal loading condition. Interestingly, as illustrated in figure 3*a*,*g*, the vein joints containing spikes display a relatively larger dorsal/ventral asymmetry than the other joints.

Loadings on the dorsal and ventral surfaces establish contacts between almost all spikes and their adjacent longitudinal veins in Model 5. Dorsal loading involves the large spikes located on the hill side of the vein joints in the deformation of the model, except for the middle joint where the small spikes on the valley side control its deformation. However, the deformation of this model under ventral loading is mainly dominated by the small spikes on the valley sides of the joints, except the deformation of the middle vein joint, which is affected by the spikes located at the hill side. The deformation of Model 5 is noticeably larger subjected to the pressure on its ventral surface.

**Figure 4.**
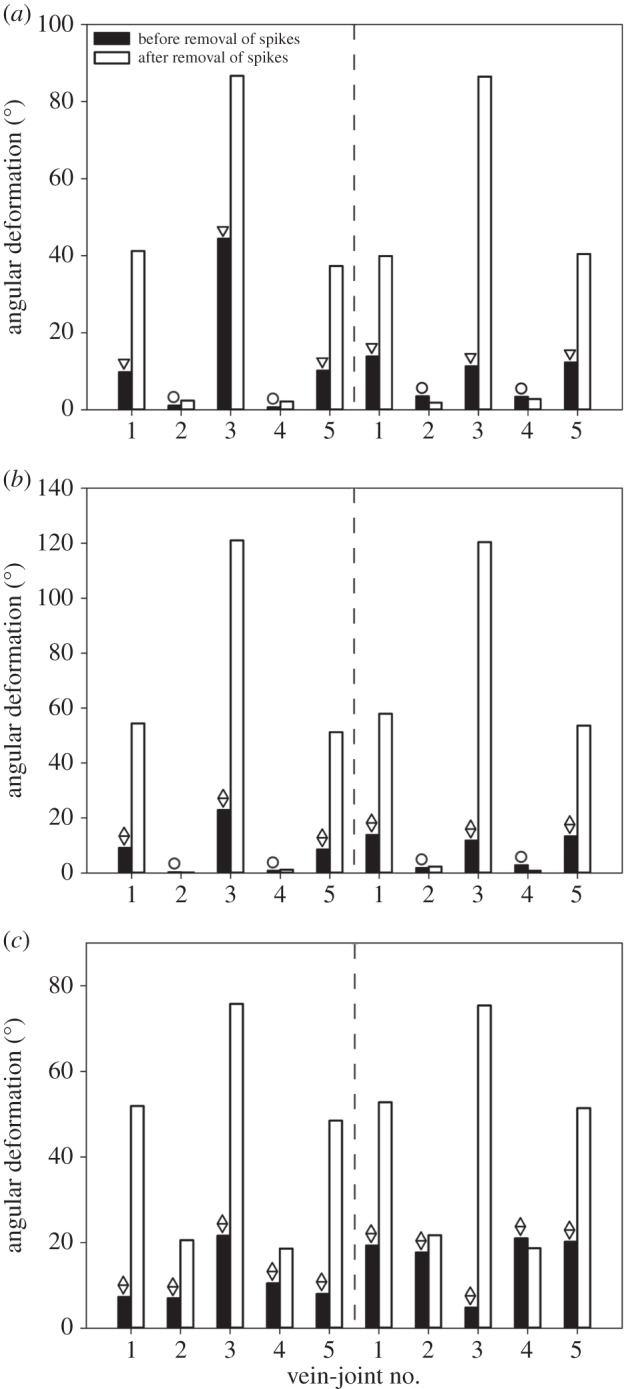
Comparison of the angular deformation of each vein joint in Models (*a*) 1, (*b*) 2 and (*c*) 3, after removal of spikes. The bar graphs on the left- and right-hand sides of dashed lines represent the angular deformations under dorsal and ventral loadings, respectively. Circles, inverted triangles and diamonds indicate the vein joints with no spike (circles), with spikes on the ventral side (inverted triangles) and with spikes on both dorsal and ventral sides (diamonds).

In order to quantify the effect of the spikes on the deformation of vein joints, we performed an additional analysis by removing the spikes located on the vein joints in Models 3–5. The results which are illustrated in figure 4 indicate a large influence of the spikes on the angular deformations of the vein joints, and therefore on the overall deformation of the models. Qualitatively, different effects were observed between the deformations of the models in the dorsal and ventral loadings (figure 4, the bar graphs on the right and left sides of dashed lines, respectively). However, after removal of spikes, the deformation of the models is almost symmetric under dorsal and ventral loadings.

**Figure 5.**
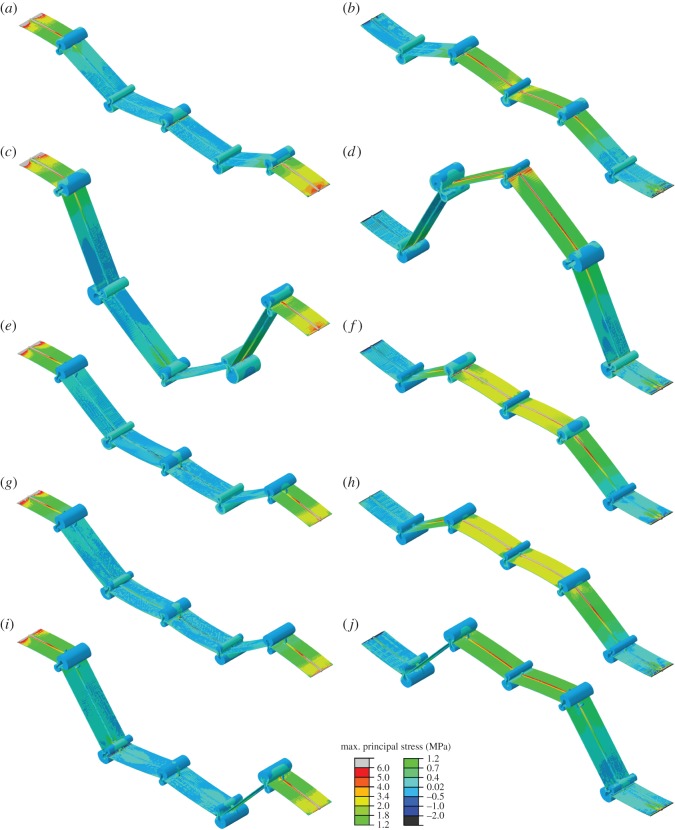
Distribution of the maximum principal stress due to a 60 Pa uniform pressure on the ventral side of vein joint combination models: (*a*,*b*) Model 1, (*c*,*d*) Model 2, (*e*,*f*) Model 3, (*g*,*h*) Model 4 and (*i*,*j*) Model 5. The pattern of the stress is presented on the (*a*,*c*,*e*,*g*,*i*) ventral and (*b*,*d*,*f*,*h*,*j*) dorsal surfaces of the models.

The distributions of the maximum principal stress in the vein joint combination models subjected to a 60 Pa pressure on their ventral surfaces are given in figure 5. Figure 5*a*,*b* represents the stress acting on the ventral and dorsal sides of Model 1 under this pressure, respectively. Taking into account that this loading condition induces bending deformations to the cross-veins and membranes connected to the middle vein joint as well as those connected to the boundary condition of this model (figure 3*h*), a high level of stress can be found in these parts. As was expected, the greatest amount of this stress is experienced by the cross-veins. There are also lower stress levels on some parts of the longitudinal veins, located close to the connection with cross-veins, which indicate the transmission of stress from cross-veins to the longitudinal veins. This stress transmission leads to relatively high stress concentrations at locations of vein joints, especially at hill-sided joints lacking resilin patches.

Although Model 2 experienced a remarkably larger overall deformation compared to all the other models and especially to Model 1, the connecting cross-veins in this model were subjected to relatively smaller bending deformations (figure 3*i*). This phenomenon leads to lower stress levels in the cross-veins in Model 2, in contrast to the cross-veins in Model 1 (figure 5). The large angular deformation of the middle vein joint in Model 2 results in a relatively high stress gradient at this joint and through the nearby membranes (figure 5*d*). However, the magnitude of the maximum stress in this joint (red colour) is still lower than the stress experienced by the surrounding flexible-fused joints (FFJR1) (grey colour) which undergo remarkably smaller angular deformations.

The stress patterns of Models 3 and 4 also indicate high stress levels on cross-veins and membranes, especially those connected to the middle joints. However, interestingly, comparison of the stress distributions at the vein joints in these two models shows much lower stress concentrations at double flexible joints (DFJS1/DFJS2) than at DFJ or at flexible-fused (FFJR1) joint. Taking into account that this loading condition (60 Pa pressure on the ventral side) establishes contacts between the spikes and longitudinal veins in the middle joints of Models 3 and 4, a stress field can be observed on the middle longitudinal veins at the connection with the spikes (figure 5*e*,*g*).

In comparison to the other models, Model 5 shows significantly lower stress concentrations at the vein joints (figure 5*i*,*j*). The occurrence of the contacts between the spikes and longitudinal veins in Model 5 causes bending of the cross-veins, which consequently leads to higher stress levels on them. However, although Model 5 experiences a larger overall deformation compared to Models 1, 3 and 4, it shows a more uniform stress distribution and a considerably lower stress level.

## 4. Discussion

### 4.1. Validation analysis

The validation analysis showed a good agreement between the experimental data [18] and our numerical results. Comparison of the results from both experiments and simulations indicated the reliability of the modelling technique and the solving procedure in the prediction of the angular deflections, and therefore the overall deformations in the dragonfly wing. Taking into account that the previous experiments were performed on whole wing samples [18], the observed difference between the results from these two methods seems to be reasonable. This difference is probably due to the higher stiffness of the real wing section which is connected to the other parts of the wing, and therefore presumably exhibits a more limited deformation in this region.

As reported by Donoughe *et al.* [18] and confirmed by our simulations, the loading condition used in the validation study (20° rotation in the clockwise direction) implies no contact between the spikes and their adjacent longitudinal veins (see the deformed shape of the model in figure 1). Considering this and the fact that this loading scenario does not allow all the vein joints to be effectively involved in the deformation, we investigated the behaviour of our models under a uniform pressure (on the dorsal and ventral sides) which is more relevant to the loading condition that an insect wing experiences during flight.

### 4.2. The effect of resilin patches on the deformation of vein joints

Taking into account that under pressures lower than a specific value (4.25 Pa on the dorsal surface and 3.00 Pa on the ventral surface) no direct contact occurs between the spikes and longitudinal veins, we use the corresponding results to study the influence of only resilin patches on the deformation represented by each joint type. Comparison of the deformations observed in different vein joints surrounded by the same joint types revealed a considerably larger angular deformation in the double-sided resilin joints in contrast to the vein joints with one-sided resilin patches or those containing no resilin (figure 2). This result indicates the important role of resilin in the enhancement of the flexibility of vein joints in dragonfly wings, as demonstrated in our previous study [25]. This effect occurs by the mechanism which allows for larger rotations of cross-veins around resilin vein joints and restricts their rotation around the joints containing no resilin. The increase in the local flexibility of insect wings obtained by resilin patches enhances their overall deformability in the chordwise direction [11,18,22]. Considering the higher flexibility of insect wings along the chord than the wing span [40], one can assume the resilin vein joints to be one of the potential sources of the flexibility differences in these two directions [18].

Our previous numerical study on isolated vein joints indicated that, under the same loading condition, one-sided resilin joints undergo significantly larger deformation than rigid joints containing no resilin. However, comparison of the mechanical behaviour of the DFJ in Model 4 (as a rigid joint containing no resilin) and the flexible-fused joints (FFJR1) in Model 3 (as a one-sided resilin joint) displayed only a slight difference between the angular deformations given by these two different vein joint types. The possible explanation for this contradiction can be found in the difference between the deformations represented by the surrounding vein joints in these two models. Interestingly, the double-sided resilin joints in Model 4 experience larger deformations in contrast to their corresponding double-sided resilin joints in Model 3 (figure 2). Taking into account that these surrounding vein joints are similar to each other (considering no spike influence), their different deformation behaviour is probably caused by the vein joints located between them. The same phenomenon was observed by the comparison of the deformation of the middle vein joints in Models 4 and 5. The double-sided resilin joints located in the middle of these two models are surrounded by DFJ in Model 4 and double flexible joints with spikes on both sides (DFJS2) in Model 5. However, these similar joints experience considerably different angular deformations under dorsal and ventral loadings. These findings suggest the influence of a vein joint on the deformation experienced by its neighbouring joints. Therefore, a more precise prediction of the deformation of insect wings at a specific joint is only possible by considering the interaction of that joint with its surrounding vein joints.

### 4.3. The effect of spikes on the deformation of vein joints

The occurrence of spikes in combination with vein joints in Odonata wings is much more common in damselflies than dragonflies [17]. The joint-associated spikes have been previously suggested to be kinds of ‘stoppers’ limiting the rotation of cross-veins around the axis of their adjacent longitudinal veins [11]. Later, by realizing that the spikes are usually combined with the vein joints containing resilin, their role in limiting the deformation of vein joints became widely accepted [18,22].

Here, by removing the spikes from the vein joints in Models 3–5, we showed how spikes can affect the deformation of the models (figure 4). The effect of the removal of spikes suggests the significant influence of spikes on the deformation experienced by all vein joints in the models. As was expected, this effect is significantly larger for the vein joints containing spikes. However, as mentioned before, the deformation of each vein joint may influence the mechanical behaviour exhibited by the surrounding joints. The dramatic increase in the deformation of some vein joints, after removal of spikes, indicates that without spikes the wings containing flexible joints would not be rigid enough to withstand mechanical forces in flight. Hence, the extremely large deformation of the models, after removal of spikes, may prevent an efficient lift generation [43].

On the other hand, the quantitatively similar deformation of the vein joints of Models 3–5, after removal of spikes, in both dorsal and ventral directions suggests that spikes may further contribute to the possible dorsal/ventral asymmetry in flexural stiffness reported by other researchers for different insect wings [40,44]. This effect may come from different shapes and sizes of spikes on the hill and valley sides of vein joints. For example, applying a 60 Pa pressure on the dorsal surface of Model 5 leads to the contact between the spikes on the valley side of the middle vein joint and the adjacent longitudinal vein (figure 3*f*). Direct physical contacts were also observed between the hill-sided spikes on this vein joint and the adjacent longitudinal vein when the model was under the same pressure on the ventral surface (figure 3*l*). Taking into account that valley-sided spikes are smaller than those located on the hill side, this vein joint shows considerably different angular deformations under dorsal and ventral loadings (figure 3*a*,*g*).

### 4.4. Stress distribution in the models

As mentioned before, the presence of resilin patches in a vein joint can effectively increase the elastic deformation under a given force (figures 2 and 3). However, as illustrated by our results, the occurrence of resilin also remarkably reduces the stress concentration in a vein joint. The considerably lower stress level in the models containing double-sided resilin vein joints (such as Model 5) suggests the important role of resilin patches in reducing the stress and providing a more uniform stress pattern (figure 5). This effect may lead to an improved ability to reduce fatigue-related damages in the wing.

It has been suggested that resilin in various parts of an insect body contributes to provide higher energy storage capacity [45]. Given the relatively large deformation of some insect wings in flight, this characteristic may prevent structural failure under repeated stresses. This conjecture can be verified by the results obtained from Model 5, indicating a noticeably low stress level, in spite of its relatively large deformation (compared to Models 1, 3 and 4; see the distribution of the stress on the dorsal surfaces of the models in figure 5).

The high stress concentrations at the rigid vein joints (or less flexible joints) and relatively lower stress levels on the longitudinal veins at the regions close to these joints indicate the transmission of the stress from cross-veins to longitudinal veins (figure 5). This stress transmission may play an important role in the stress distribution throughout the parts of insect wings which mainly consist of rigid or less flexible vein joints. Taking into account that longitudinal veins are usually bigger in size and they probably show higher resistance to the applied stresses than cross-veins, this phenomenon may further avoid the material failure in cross-veins.

### 4.5. Possible influence of vein joint combinations on camber formation

The flexible wings of most damselflies are good examples of wings with the ability of automatic camber formation. Based on the mechanism explained by Ennos [46], in a corrugated wing containing spars which are branched from the leading edge and move towards the trailing edge, the torsion of the wing leads to camber generation. However, as illustrated by our models, the quantity of this cambering is highly dependent on the combination of different joint types in the wing.

According to our numerical results, although the presence of resilin patches enhances the flexibility of the models, they contribute to camber formation only when they are located on both hill and valley sides of vein joints (Models 2–5). In this case, these double-sided resilin joints may develop a cambered section even if they are combined with rigid (Model 4) or less flexible (Models 2, 3 and 5) vein joints.

Our results showed that, although Models 2–5 exhibit different magnitudes of deformation, the cambering in all of them occurs via the same mechanism. Under a pressure applied to the ventral side, the vein joint located in the middle of each model is raised and causes the adjacent longitudinal veins to rotate around their axes. This deformation in real flight may push down the trailing edge, and therefore results in camber formation [46,47].

In comparison with damselflies, the wings of dragonflies show less twisting capability [8]. It was suggested that these different torsional deformations may come from the different positions of nodus in wings of dragonflies (between 47 and 60% of the forewing's length and 40–46% of the hindwing's length) and damselflies (between 29 and 37% of the length of both wings) [8]. However, based on our numerical results, one can conclude that this difference may arise either from different vein joint combinations in wings of these two suborders of Odonata. The wings of many dragonflies are dominated by flexible-fused vein joints (FFJR1), but, in damselflies, wings are characterized by combinations of double flexible joints, which may lead to a strong tendency of camber formation. As previously stated, the lack of double-sided resilin vein joints in broad-based wings of some dragonflies is presumably counterbalanced by the presence of posterior-distally curved longitudinal veins and polygonal cells facilitating camber development [11].

It is also important to note that the large-scale deformation observed in Group 2 of vein joint combinations (Model 2) seems to be a reasonable result. The absence of spikes in the wings belonging to this group is correlated with the presence of double-sided resilin joints (DFJR2). These two factors together may result in large deformations which are more likely to happen in relatively large wings of *A. cyanea*, *P. apicalis* and *T. pryeri*. However, experimental studies are necessary to evaluate the camber formation in the wings of these species.

## 5. Conclusion

In this article, using a series of numerical simulations, we showed that different vein joint combinations may significantly influence deformations experienced by insect wings, and therefore their flight performance. The developed models, which enabled us to simulate the camber in insect wings, suggest the crucial role of double-sided resilin joints in camber formation. We further used the models to obtain experimentally inaccessible data, such as the stress distribution in veins and membranes. The findings indicate that the presence of a series of elastic elements, resilin patches, at the connection of longitudinal veins and cross-veins may significantly increase the deformability of whole wing structure. Resilin-dominated vein joints may further contribute to a more uniform stress distribution and considerably lower stress levels within insect wings. On the other hand, spikes located at vein joints act as stoppers which mechanically limit their angular deformations, and therefore prevent the structural damage and aerodynamic instability due to extremely large deformations. Based on the numerical results, the spikes may further contribute in the dorsal/ventral asymmetry of wing deformations.

## Supplementary Material

Figure S1: Example micrographs of main joint types in Odonata wings. All images were taken from S. vulgatum forewing. SEM images of (a) DRFJ and (b) DFJ (both from the posterior-distal region), (c) the valley side of BJ located at ScP, (d) the fused side and (f) the flexible side of two flexible-fused joints with a resilin patch at the valley side (FFJR1) located at RA, (h) the valley side of FFJR1 from the basal part, (i) the hill side and (j) the valley side of two double flexible joints with spikes on the hill side (DFJS1), (k) the valley side and (l) the hill side of a double flexible joints with spikes on both sides (DFJS2). The blue colour indicates the presence of resilin in the vein joints. Microscopic images of (e) a fused vein joint taken by wide-field fluorescence microscope and (g) a flexible vein joint imaged by CLSM (from Appel and Gorb, 2014). The light blue colour in the CLSM image is due to the blue autofluorescence characteristic of resilin under ultraviolet (UV) light excitation. Scale bars: (a), (b), (e), (f), (g), (h), (i), (k), (l) 50 μm, (c), (d), (j) 100 μm.

## Supplementary Material

Figure S2: Five groups of vein joint combinations. (a, c, e, g, i) Schematic wing cross sections from Groups 1-5. (a) Wings of representatives of Group 1 are dominated by flexible-fused joint combinations with resilin patches at the flexible joints. (c) Wings of representatives of Group 2 are dominated by flexible-fused joints, but show an increased number of double flexible joint combinations. (e) Wings of representatives of Group 3 show flexible-fused and double flexible joint combinations, the latter with spines on the hill-sided joints. (g) Wings of representatives of Group 4 show an alternating distribution pattern of longitudinal veins either dominated by double fused or double flexible joint combinations, with the latter bearing spines on both sides. (i) Wings of representatives of Group 5 are dominated by double flexible joint combinations with spines on both flexible sides. (b, d, f, h, j) Wings of representatives of Groups 1-5. (b) E. superstes, (d) P. apicalis, (f) S. vulgatum, (h) P. Romulus, and (j) M. pruinosa pruinosa. The red rectangles in this figure correspond to the approximate areas on wings, where these vein joint combinations may occur (Reconstructed by permission from Appel and Gorb, 2014).

## Supplementary Material

Model S1: The FE model of vein joint combination Group 1. Model S2: The FE model of vein joint combination Group 2. Model S3: The FE model of vein joint combination Group3. Model S4: The FE model of vein joint combination Group 4. Model S5: The FE model of vein joint combination Group 5.

## Data accessibility

All supporting data are made available either in the article or the electronic supplementary material.
