# Supplementary material for: Effects of multiple vein microjoints on the mechanical behaviour of dragonfly wings: numerical modelling
Source: R Soc Open Sci. 2016 Mar 23;3(3):150610. doi: 10.1098/rsos.150610 (PMC4821260; doi:10.1098/rsos.150610)
Supplement: Figure S2: Five groups of vein joint combinations. (a, c, e, g, i) Schematic wing cross sections from Groups 1-5. (a) Wings of representatives of Group 1 are dominated by flexible-fused joint combinations with resilin patches at the flexible joints. (c) Wings of representatives of Group 2 are domina [file rsos150610supp2.pdf]

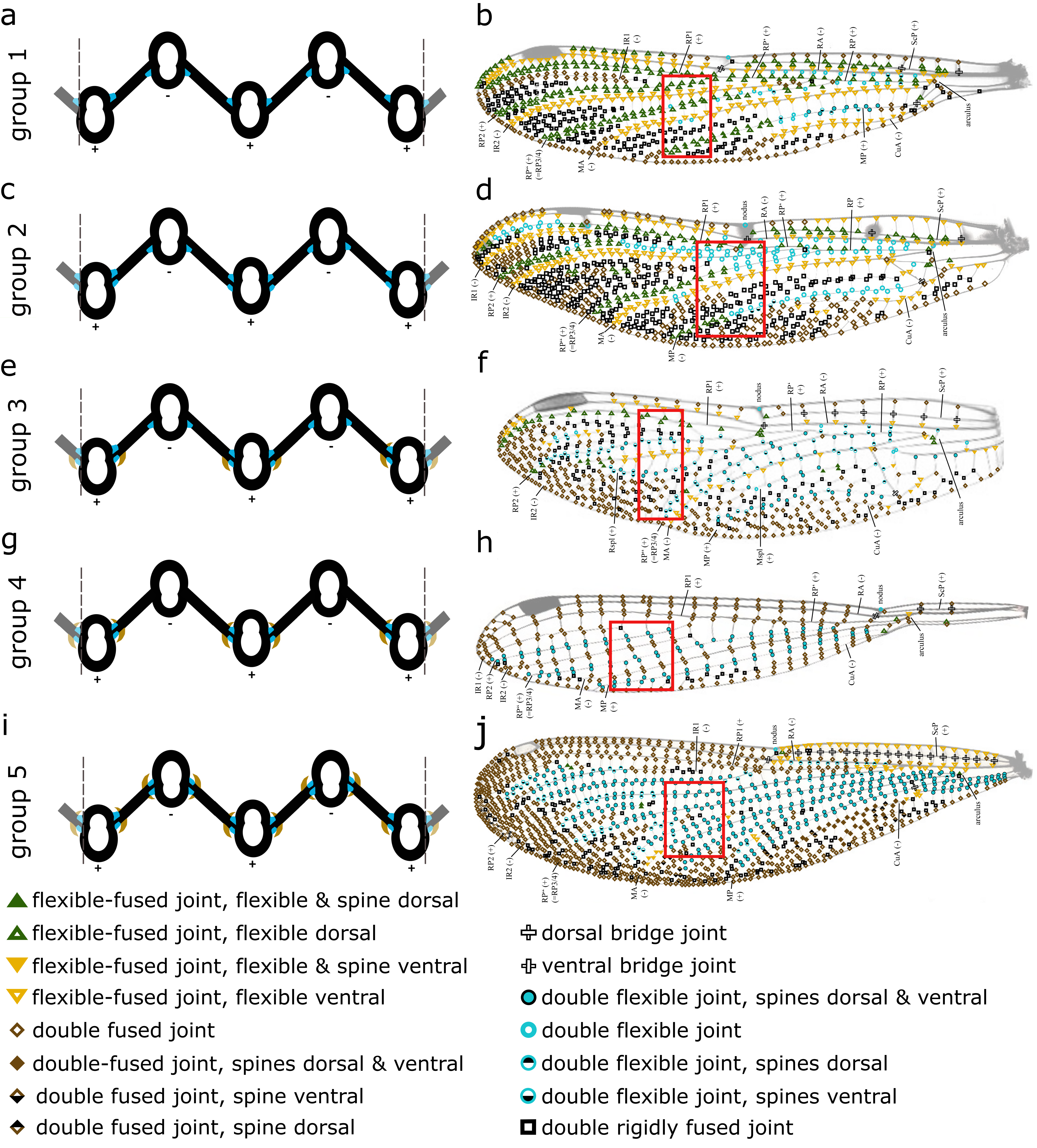
a
b
c
d
e
f
g
h
i
j
group 1
group 2
group 3
group 4
group 5
flexible-fused joint, flexible & spine dorsal
flexible-fused joint, flexible dorsal
flexible-fused joint, flexible & spine ventral
flexible-fused joint, flexible ventral
double fused joint
double-fused joint, spines dorsal & ventral
double fused joint, spine ventral
double fused joint, spine dorsal
dorsal bridge joint
ventral bridge joint
double flexible joint, spines dorsal & ventral
double flexible joint
double flexible joint, spines dorsal
double flexible joint, spines ventral
double rigidly fused joint
